# Coping Strategies and Family Crisis Among Relatives of Intensive Care Unit Patients

**DOI:** 10.1192/j.eurpsy.2025.484

**Published:** 2025-08-26

**Authors:** Z. Konstanti, M. Kourakos, F. Tatsis, S. Georgakis, G. Papathanakos, M. Gouva, V. Koulouras

**Affiliations:** 1 Department of Nursing, School of Health Sciences, University of Ioannina, Research Laboratory Psychology of Patients, Families & Health Professionals; 2 Department of Intensive Care Medicine, University Hospital of Ioannina, Ioannina, Greece

## Abstract

**Introduction:**

The admission of a patient to the Intensive Care Unit (ICU) often triggers intense stress symptoms in their family members, leading to anxiety, depression, and emotional distress.

**Objectives:**

To investigate the coping mechanisms employed by family members of critically ill patients, considering their sociodemographic characteristics and the closeness of their relationship to the patient.

**Methods:**

The study included first-degree relatives, close relatives, and intimate friends of ICU patients. Data were collected through written questionnaires completed by family members who visited patients during the first week of treatment. A sociodemographic questionnaire and the Family Crisis Oriented Personal Evaluation (F-COPES) scale were used to assess coping strategies.

**Results:**

A total of 223 family members, with a mean age of 41.5 ± 11.9 years, participated in the study. This sample represented 147 critically ill patients. Of the participants, 81 (36.3%) were men and 142 (63.7%) were women. The majority were the patients’ children (40.8%), siblings (19.3%), or companions (16.1%). The most frequently employed coping strategies were seeking social support (mean score 31.27 ± 4.72), mobilizing the family for help (mean score 14.26 ± 3.17), and adopting a passive approach (mean score 14.04 ± 2.81). Offspring and male relatives were less effective in using coping strategies compared to other relatives and female participants, respectively.

**Conclusions:**

Family members of critically ill patients are psychologically vulnerable, experiencing significant emotional distress. They often rely on external support to cope with the family crisis. Health professionals should prioritize understanding and addressing the specific needs of these relatives to provide appropriate support.

**Disclosure of Interest:**

None Declared

